# Relative advantages of dichromatic and trichromatic color vision in camouflage breaking

**DOI:** 10.1093/beheco/arw185

**Published:** 2017-02-04

**Authors:** Jolyon Troscianko, Jared Wilson-Aggarwal, David Griffiths, Claire N. Spottiswoode, Martin Stevens

**Affiliations:** a University of Exeter, School of Biosciences, Penryn Campus, Penryn TR10 9FE, UK,; b FoAM - Kernow, Workshop E, Jubilee Warehouse, Commercial Road, Penryn TR10 8FG, UK,; c University of Cambridge, Department of Zoology, Downing Street, Cambridge CB2 3EJ, UK, and; d DST-NRF Centre of Excellence at the FitzPatrick Institute, University of Cape Town, Rondebosch 7701, South Africa

**Keywords:** background matching, camouflage, citizen science, color vision, learning, polymorphic trichromacy, sensory ecology.

## Abstract

There is huge diversity in visual systems and color discrimination abilities, thought to stem from an animal’s ecology and life history. Many primate species maintain a polymorphism in color vision, whereby most individuals are dichromats but some females are trichromats, implying that selection sometimes favors dichromatic vision. Detecting camouflaged prey is thought to be a task where dichromatic individuals could have an advantage. However, previous work either has not been able to disentangle camouflage detection from other ecological or social explanations, or did not use biologically relevant cryptic stimuli to test this hypothesis under controlled conditions. Here, we used online “citizen science” games to test how quickly humans could detect cryptic birds (incubating nightjars) and eggs (of nightjars, plovers and coursers) under trichromatic and simulated dichromatic viewing conditions. Trichromats had an overall advantage, although there were significant differences in performance between viewing conditions. When searching for consistently shaped and patterned adult nightjars, simulated dichromats were more heavily influenced by the degree of pattern difference than were trichromats, and were poorer at detecting prey with inferior pattern and luminance camouflage. When searching for clutches of eggs—which were more variable in appearance and shape than the adult nightjars—the simulated dichromats learnt to detect the clutches faster, but were less sensitive to subtle luminance differences. These results suggest there are substantial differences in the cues available under viewing conditions that simulate different receptor types, and that these interact with the scene in complex ways to affect camouflage breaking.

## Lay Summary

Online camouflage games reveal trichromats are better at finding birds and eggs than simulated dichromats, but dichromats can learn faster. Color vision varies markedly between species, such as between dichromats with 2 different color receptors, and trichromats with 3. Contrary to expectation, trichromats captured the nightjars and eggs faster than simulated dichromats, but simulated dichromats' capture times were affected less by a number of camouflage variables, and they learnt to capture eggs faster.

Twitter: @SensoryEcology #colsci

## INTRODUCTION

Animals exhibit a striking diversity of visual systems among taxa, species, and sometimes even within the same species. Although the selection pressures and physiological constraints that cause such differences are poorly understood, at least some appear to stem from selection for specific tasks, such as mating or foraging (Stevens 2013; Cronin et al. 2014). For example, *Heliconius* butterflies and some fireflies have visual systems tuned to detect the mating signals of conspecifics (Cronin et al. 2000; Briscoe et al. 2010), and deep-sea fish have visual pigments sensitive to their own species-specific bioluminescent light spectra (Douglas et al. 2000). One of the main differences observed in vision is in the ability to perceive color; i.e. having 2 or more receptor types that are used to discriminate different parts of a visual spectrum (Kelber et al. 2003). Color vision varies greatly among animals, from monochromatism (no color vision; e.g., some marine mammals), dichromatism (2 receptors involved in color vision; e.g., most mammals), trichromatism (e.g., humans, some fish, and bees), to tetrachromatism (e.g., many birds) and even more receptor types in some invertebrates (Cronin et al. 2014). Understanding why such diversity exists is a major challenge in visual ecology.

Primate species that have a polymorphism granting some individuals trichromatic color vision, and others dichromatic color vision, have offered an evolutionary model to test color vision hypotheses. Humans with normal vision are examples of trichromats, possessing longwave (LWS), mediumwave (MWS), and shortwave (SWS) sensitive cone types, and these are used to create 2 opponent color channels (red–green by comparing LWS to MWS, and blue–yellow by comparing the combination of LWS and MWS to SWS, Kelber et al. 2003). Dichromats lack either the LWS or MWS cone type, meaning they have no red–green opponent channel. Perceived brightness, or “luminance” in trichromats is the combination of LWS and MWS cones, or just the MWS or LWS in dichromats (Sharpe et al. 2005). This luminance channel is thought to be used for the detection of pattern, general spatial information in a visual scene, and movement (Osorio and Vorobyev 2005). The persistence of a stable polymorphism for both trichromats and dichromats in platyrrhines and prosimians, which may have persisted for up to 14 million years (Surridge and Mundy 2002), suggests that there must be some adaptive advantage to dichromatic vision, although the evolutionary forces remain hotly debated. Trichromatic vision has been demonstrated to have a number of advantages, such as in detecting red fruit and leaves against green foliage (Mollon 1989; Caine and Mundy 2000; Dominy and Lucas 2001; Osorio et al. 2004; Hiramatsu et al. 2008; Melin et al. 2013), and finding these red targets under fluctuating lighting conditions (Mollon 1989; Lovell et al. 2005). In addition, trichromats have been shown to be better able to detect sexual signals (Changizi et al. 2006) and predators (Pessoa et al. 2014). Any selective advantages of dichromatic vision are less well understood, but camouflage breaking (i.e., the detection of camouflaged objects) is thought to be a major driving force. The hypothesis that reduced color sensitivity could aid camouflage breaking has long been suggested in humans (Nature Editorial 1940), and this idea has since gained empirical support. For example, when searching for a camouflaged target, human dichromats outperform trichromats when color is irrelevant to the task (Morgan et al. 1992; Saito et al. 2006), and this is paralleled in captive brown capuchins (*Cebus apella*) and long-tailed macaques (*Macaca fascicularis*) (Saito et al. 2005). However, the stimuli used in these tasks were stylized, geometric shapes with extreme colors and little natural variation in background appearance or prey appearance compared to real cryptic prey in the wild. Some field evidence also supports a dichromat advantage; for example, dichromatic monkeys eat a larger number of cryptic insects than do trichromatic individuals of the same species (Melin et al. 2007; Smith et al. 2012). Yet, there is no evidence that dichromats spend more time searching in niches associated with cryptic prey than in niches associated with colorful food resources (Melin et al. 2008), and trichromats may capture more prey overall (Smith et al. 2012). Thus, the evidence in support of this hypothesis remains equivocal.

Modeling by Osorio et al. (2004) predicted that dim lighting conditions should favor trichromats when searching for fruit, because cones sensitive to short wavelengths have a lower photon catch than the more abundant cones sensitive to medium and long wavelengths. This in turn means the blue–yellow opponent channel (the only channel available to dichromats) would be subject to more noise than the red–green opponent channel under low light conditions. However, a number of field experiments on nonhuman primates suggest a dichromat advantage in low daylight conditions. For example, females in 2 polymorphic monkey species (*Propithecus v. verreauxi* and *Ateles geoffroyi*) were found to preferentially feed at higher light intensities than their solely dichromatic male conspecifics (Yamashita et al. 2005). Dichromatic capuchins (*Cebus capucinus*) have also been found to make more prey capture attempts in the shade than trichromatic conspecifics (Melin et al. 2007), and dichromats were found to outperform trichromats when foraging in the shade, even though they did not spend more time searching in the shade (Caine et al. 2010). Thus differences in behavior (e.g., foraging strategies) and ecology may confound effects of visual system performance, and field experiments cannot easily control for ecological and behavioral factors that might correlate with lighting conditions, such as different prey species inhabiting the shade at the bottom of the canopy, activity changes with time of day or weather, or social/antipredator factors that might affect the canopy height usage of males and females.

Experiments under controlled conditions are perhaps the most effective means to compare the performance of subjects with dichromatic and trichromatic color vision in finding camouflaged targets or ripe fruit. A useful experimental technique is to present one type of receiver with a visual scene modified to simulate the visual information available to receivers with different visual systems, since experimentally modifying the receiver’s visual system itself is impractical. In such computer-based experiments, humans searching for fruit against a green foliage background found red, orange, and yellow fruits faster when viewing images in trichromatic vision than when images were modified to represent dichromatic vision. However, trichromatic advantage was less clear with dark or purple fruit, suggesting that luminance cues in dark targets could have given dichromats more salient information (Melin et al. 2013). Thus, lighting conditions and achromatic cues are likely to be important factors maintaining dichromatic vision, but how these affect camouflage breaking is not understood. Melin et al. (2013) demonstrate the value of using simulated human dichromatic and trichromatic visual search tasks to approach this question. However, their experiment focused on detecting fruit, which have evolved to attract visually guided frugivores by contrasting with the background color. Cryptic prey provide a qualitatively different search task, involving matching both the color and pattern of the background, or using edge disruption to break up the prey’s outline (Stevens and Merilaita 2009). Moreover, to our knowledge previous experiments have only considered the performance differences between visual systems, and have not attempted to quantify the camouflage of each stimulus and how this interacts with differences between visual systems.

Here, we assessed the camouflage-breaking abilities of humans under trichromatic and simulated dichromatic conditions in an ecologically relevant search task, using photographs of highly cryptic adult nightjars and ground nesting plover/courser nests (eggs). These are known to be preyed upon by both dichromatic and trichromatic predators (Troscianko et al. 2016b). We used a “citizen science” (Bonney et al. 2014) computer-based detection task with human observers. Computer displays are a useful tool for investigating visual properties that affect detection times (Melin et al. 2013, Stevens et al. 2013, Troscianko et al. 2013). Our aim was to determine which visual system (trichromacy, or simulated dichromacy, referred to as “viewing condition” hereafter) performed better at finding camouflaged prey when under a time limit, and how the prey camouflage and background features affected relative success. Additionally, we investigated whether changing the viewing condition colors affected the ability of participants to learn to break camouflage, as learning can affect performance over repeated encounters differently, depending on camouflage type and contrast (Troscianko et al. 2013). On the basis of the studies discussed above, we might expect simulated dichromats to perform better at finding targets that have a lower overall luminance than their backgrounds, or to perform better where pattern and contrast cues are required to break the camouflage. If color information is less informative than such contrast, textural or pattern cues, then we might also expect the simulated dichromats to learn to find prey faster, in line with previous work (Troscianko et al. 2013), as color information will offer less of a distraction (Morgan et al. 1992; Saito et al. 2006). However, given that the majority of participants’ visual systems will be accustomed to using trichromatic vision, there may also be a learning difference when they are subject to a simulated dichromat scene when adjusting to the loss of color information.

## METHODS

**Figure 1:**
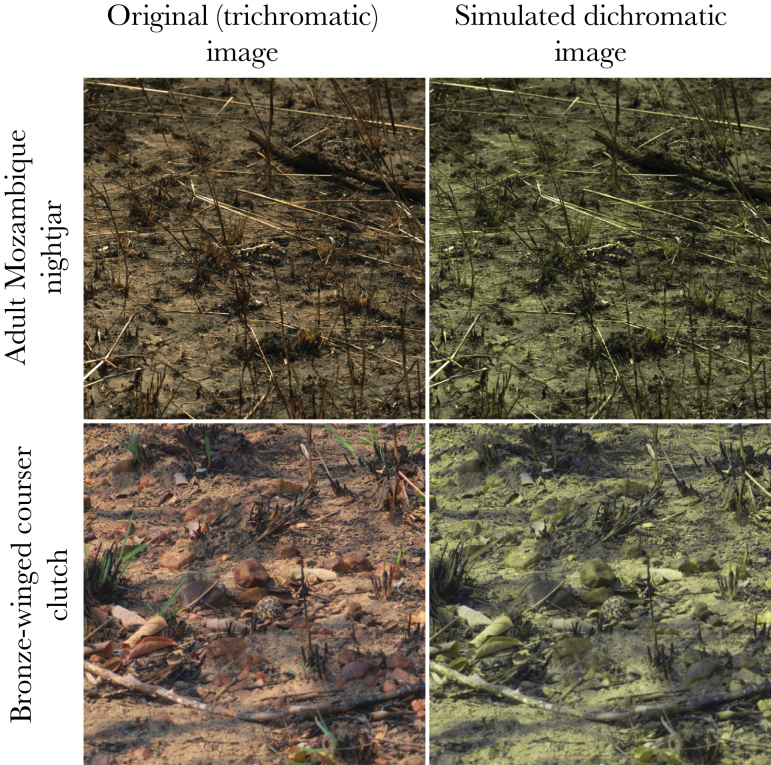
Sample images used in the online game in trichromatic (left column) and simulated dichromatic colors (right column). These images show a 1500 by 1500 pixel crop of the full image.

We created 2 online “citizen science” games that were played over the internet by members of the scientific community and general public. The games (full details below) were loaded in a web browser and comprised of a sequence of photographs of camouflaged real animals from the wild, with participants being asked to detect them (by clicking on them) as quickly as possible. Each game was played in one of 2 versions, either in trichromatic color vision, or simulated dichromatic vision (Figure 1 shows sample images). Experiment 1 involved participants searching for camouflaged adult birds (nightjars) sitting on nests, whereas experiment 2 involved subjects searching for camouflaged birds’ eggs.

### Photographs

The games used photographs of nesting birds or eggs from field sites in Zambia and South Africa (Figure 1). Nests were located in an area of c. 3100 ha around Musumanene and Semahwa farms (centered on 16°46’S, 26°54’E), and c. 400 ha on Muckleneuk farm (centered on 16°39’S, 27°00’E), in the Choma District of Zambia, during September–November 2012–2013, and on the salt pans and beaches near Velddrif, South Africa (centred on 32°48’S 18°10’E) during August–September 2013. Nests in Zambia were principally located by local farm laborers as the birds flushed on approach of the searchers or their cattle, and some nightjars were located through nocturnal eye-shine. Nests in South Africa were on pebble beaches or the silt and rock edges of salt pans, found by walking slowly along the linear habitats and using binoculars to identify birds flushing from their nests.

For experiment 1, photographs of adult nightjars (*n* = 39, Caprimulgiformes, Caprimulgidae: fiery-necked nightjar *Caprimulgus pectoralis* [8], Mozambique nightjar *Caprimulgus fossii* [26], and pennant-winged nightjar *Macrodipteryx vexillaria* [5]) were taken from a distance of 5 m using a Nikon D7000 camera with a Nikon 105 mm lens. Adult nightjars were always photographed from their most visible flank, or if both sides were unobstructed then photographs were taken pointing away from the sun to minimize lens flare. White balance in each image was set from a gray standard photographed after each nightjar using the same camera settings. For experiment 2, we used plover and courser eggs (*n* = 88, Charadriiformes, Charadriidae: crowned plover *Vanellus coronatus* [40], wattled plover *Vanellus senegallus* [5], 3-banded plover *Charadrius tricollaris* [3], white-fronted plover *Charadrius marginatus* [6], chestnut-banded plover *Charadrius pallidus* [2] and blacksmith plover *Vanellus armatus* [1]; Charadriiformes, Glareolidae: bronze-winged courser *Rhinoptilus chalcopterus* [21] and Temminck’s courser *Cursorius temminckii* [10]), which were photographed in both Zambia and South Africa. All photographs were taken in direct sunlight and not within 2 h of sunrise or sunset to keep images standardized. All trichromatic photographs were also converted into dichromatic images, whereby the red and green channels were combined (Y = (R + G)/2), to create a blue–yellow image (see Figure 1). Rather than attempting to match any specific visual system or color vision abnormality (which would not be accurately reproduced on uncalibrated computer monitors used by game participants), we chose to use the combination of the camera’s red and green channels as an approximation of how a scene would be viewed by a general mammalian dichromat with longwave and shortwave cone types. This approximation uses broader-band longwave sensitivities than would be found in a typical opsin response; however, by using measures of luminance that are identical in both viewing conditions, this approach facilitates more direct comparisons between the luminance-based camouflage metrics under the different viewing conditions (see below). Moreover, the backgrounds in our dataset contained very few bright red or green objects, since they were mostly brown; this indicates that choice of red and green combined would be qualitatively similar to using red or green instead. Survival data from Zambian fieldwork demonstrated that the nightjar, plover, and courser nests were preyed upon by both trichromatic and dichromatic predators (vervet monkeys and mongooses respectively, Troscianko et al. 2016b), implying that these species’ camouflage is likely to have evolved under selection pressures from similar visual systems.

Camouflage metrics were measured from the luminance channel of each photograph, calculated as the average of red and green channels used for human luminance processing (Sharpe et al. 2005). Note that this is not a measure of absolute luminance or radiance reaching the viewers’ eyes, because the varied displays used to play the game will all have had different properties, and each participant’s vision will adapt to their own display. This source of error is controlled for in the mixed model statistics, accounting for differences between sessions (see below). Hereafter the term luminance therefore refers to the relative red and green pixel brightness of a display, limited by the display’s unknown dynamic range, nonlinearity and absolute luminance. The achromatic luminance channel is thought to be used in pattern detection (Osorio and Vorobyev 2005), and means that measurements were identical in both the trichromatic and dichromatic versions of each image. The target in each image (clutch or adult nightjar) was selected using a polygon tool in ImageJ. Two areas of background were then measured. First, a surrounding “doughnut” shaped region between a radius of 15 px (pixel) and 500 px from the target edge was selected. This area comprised just the region surrounding the target, the majority of which would be visible wherever the target was randomly positioned on viewers’ screens (see below). The region within 15 px of the target was excluded to ensure that no out-of-focus regions in front of the target were measured (as these would be a combination of both target and parts of the environment). The second measured region was the entire background minus the target and 15 px spacer. Contrast within each patch was the standard deviation of luminance values within each patch, divided by the mean. Pattern and luminance differences were calculated between the target and the 2 respective backgrounds using our Multispectral Image Analysis and Calibration Toolbox (Troscianko and Stevens 2015). Pattern was measured using granularity analysis, a widely used approach for measuring the contrast of patterns at different spatial scales thought to resemble the early processing of visual signals in many species (Godfrey et al. 1987; Chiao et al. 2009; Stoddard and Stevens 2010). Pattern difference metrics followed Troscianko et al. (2016b) and were generated using 15 spatial granularity bands from 2 px to 256 px (adult nightjar game), or 13 bands from 2 px to 128 px (egg game), incrementing at multiples of √2. Maximum band sizes were limited to the sizes of the smallest adult nightjar or egg target in each game. “Luminance distribution difference” is a nonparametric measure of overlap between the target’s luminance histogram and the background’s luminance histogram, which can account for non-normally distributed luminance values common in animal patterning (see Troscianko et al. 2016b). These were based on measurements of 100 histogram bins. Although the photographs were standardized, we were unable to control for the end user’s visual display properties because the game was internet-based. Nevertheless, differences in display settings would only be expected to add noise to our dataset rather than any systematic bias between dichromatic and trichromatic photograph presentation. The large sample sizes of subjects (see below) should, to a large extent, overcome such noise.

### Online computer games

The online games were freely playable on internet browsers, created using custom written Scheme code compiled to JavaScript (the source code and files are all available: Griffiths 2015a, 2015b). Subjects were recruited via social media, online news stories, and word of mouth. Prior to playing, the games asked participants to consent to their data on game performance (i.e., detection times) being used for scientific purposes; to give their age bracket (<10, 10–15, 16–35, 36–50, >50, measured to control for any age effects); to state whether they had played the game before; and to choose whether they would like to play as a simulated dichromat (“mongoose” for the nightjar game; “genet” for the egg game) or a trichromat (“vervet monkey” or “baboon”, respectively). Additionally, in experiment 2, the egg game, participants could choose between “easy” or “difficult” eggs, which were previously categorized subjectively by the authors based on their level of difficulty. This was done because some of the clutches were deemed too difficult to keep naïve participants playing the game until the end. Participants were only classed as naïve the first time they played each game (adult nightjar game or egg game) irrespective of the viewing conditions or difficulty level previously selected. Participants were then asked to click on the nightjar or eggs as soon as they saw them. The location of the target was made random in each slide without touching the edges of the screen by shifting the photograph on the screen, ensuring the target was placed within a central area defined by the middle 60% of the image width/height. When participants successfully clicked on the region covered by the target, their capture time was displayed and they would progress to the next slide upon clicking a message. Participants were given 30 s to find the target in each slide. If they failed to find it after that time, their data were discarded because we were unable to determine whether this represented a genuine failure to find the target, or whether the participant was distracted from the game temporarily. If participants failed to find the bird or clutch, a message stated that they had run out of time and a box was drawn around the object to show its location, and the subject could then move onto the next slide. All click coordinates were recorded (including false positives) to the nearest 1000th of a second. A total of 20 randomly selected slides were presented, and each participant’s mean capture time was displayed at the end.

### Statistics

Data were filtered to exclude any slide where participants had more than 2 incorrect clicks; this excluded trials where subjects used a “scatter-gun” strategy that might have allowed fast capture times by randomly clicking the screen repeatedly without the participant identifying the target. Subjective assessment of people’s behavior when playing (for example while interacting with participants at science festivals) suggests they switch from an efficient search (target “popping out”) to inefficient search (slow, scanning) in the first few seconds (Troscianko et al. 2009), then may resort to “scatter-gun” clicking at the screen when the time is running out. Normal search behavior then resumed with the next slide (i.e., participants did not continue to use the “scatter-gun” strategy). Successful capture times were log-transformed to create a normally distributed response error. Regrettably the online games did not record the images associated with timeout events, and as such we were unable to use survival statistics. Survival statistics could offer a more robust means for dealing with timeout events by considering the survival to the point of censorship. However the nature of online games means we cannot be certain that the participant was searching for the target, or whether they were distracted. Censoring in survival statistics would not overcome these events (which are different to people searching for and not finding the target), as such we chose to use linear statistics and exclude timeout events. If enough participants were failing to find the targets this would create bounding and heteroscedasticity in the model residuals. However, there was no apparent bounding caused by the 30-s cutoff, suggesting this would not affect the results, and the data were also checked for overdispersion. The following fixed effects were used in maximal models for capture times: slide number (also modeled as a polynomial to allow for tail-off in learning rates), trial naïvety (i.e., the first slide was treated as categorically different from subsequent ones; Troscianko et al. 2013), participant naïvety (whether the participant had played the game before), edge distance (the distance between the target and the nearest screen edge), participant age class, viewing condition (dichromatic or trichromatic images), average target luminance, target contrast, target area (size, in pixels), background luminance and contrast, pattern difference, and luminance distribution difference. In addition, the following random effects were fitted: session ID, photograph ID, and season (egg data only). All data collected were anonymous; identifying information (such as IP address) was not used to identify individuals as this is unreliable for users behind private networks. Thus, we relied on users stating whether they had played before to account for learning effects in repeat players, and to control for nonindependence to some degree. Maximal models containing all terms were fitted with either the 500 px surround comparisons or whole image comparisons, and the model with the better fit was used. Covariance between fixed effects was checked in a Spearman covariance matrix (Zuur et al. 2009). Models were checked for homogeneity of variance and normal error distributions (resulting in a log-normal model); pattern difference was also log-transformed to meet these assumptions. The maximal linear mixed effects model was specified using LMER (package lme4 version 1.0–6; Bates et al. 2014) in R version 3.0.1 (R Core Team 2013), with all 2-way interactions between the camouflage-based fixed effects and image sequence number (to account for learning rates). These models were then simplified using BIC (Bayesian information criterion)-based model selection. BIC is similar to the AIC (Akaike information criterion) method for choosing models; however, it places a greater penalty on more model terms than AIC, making over-fitting less likely. Model fitting first involved backwards fitting the fixed effects in maximum likelihood models, then forward fitting the random effects, and re–back-fitting the fixed effects using fitLMER.fnc (package LMERConvenienceFunctions version 2.5; Tremblay et al. 2013). For full and simplified model terms see the Supplementary Material. Lower estimates of degrees of freedom are reported with F-statistics.

## RESULTS

### Experiment 1: nightjars

**Figure 2:**
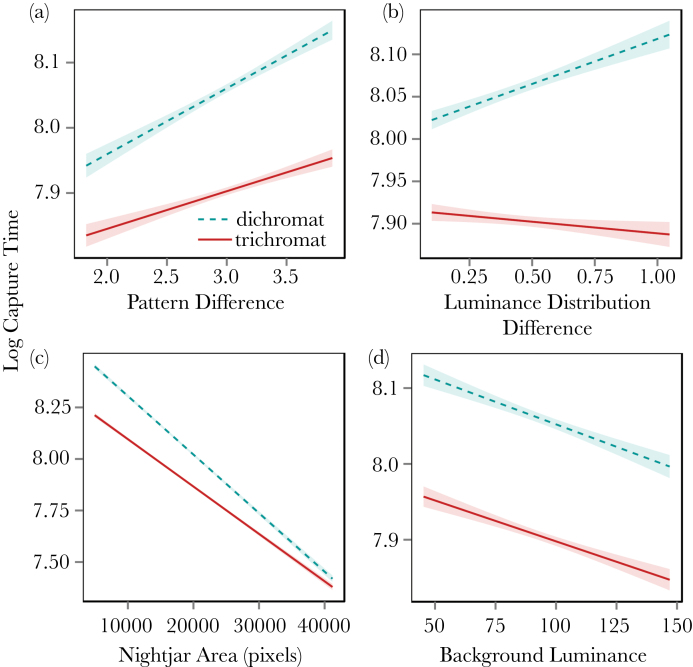
Plots showing the camouflage variables that were found to be affected by trichromatic or simulated dichromatic viewing conditions in the adult nightjar searching game (*P*-values for all 4 interactions <0.005). Overall, trichromats have an advantage over dichromats in all circumstances. However, simulated dichromats performed comparatively less poorly with lower pattern difference values (panel a), lower luminance distribution differences (panel b), and smaller targets (panel c); but not with targets positioned on higher luminance backgrounds (panel d). Lines are regressions from raw data, and shaded regions show standard error.

A total of 135968 successful nightjar “captures” were recorded from 9926 game plays (4842 naive players). On average, trichromats found the nightjars faster than simulated dichromats under all circumstances. The simplified model contained 4 interactions that all involved viewing condition as follows: 1) both trichromats and simulated dichromats took longer to detect the targets when their pattern difference compared to the background was higher; however, simulated dichromats were significantly more influenced by pattern difference than were trichromats (*t* = −12.21, *F*_1,125985_ = 82.0, *P* < 0.005, i.e. the slope for dichromats in Figure 2a is steeper than that for trichromats). 2) Simulated dichromats were more likely to take longer to detect targets than were trichromats as the luminance distribution difference increased (*t* = −11.92, *F*_1,125985_ = 111.8, *P* < 0.005). As an example of effect size, prey in the highest 10^th^ quantile of luminance distribution differences, had a predation risk 23% greater when viewed as a trichromat than when viewed as a simulated dichromat based on the raw data (24% based on model predictions). 3) Simulated dichromats also had higher capture times with smaller targets (*t* = 21.3, *F*_1,125985_ = 410.7, *P* < 0.005), and 4) when backgrounds were darker (*t* = 11.43, *F*_1,125985_ = 130.7, *P* < 0.005), see Table 1, Figure 2 and the Supplementary Statistical Output. There was no evidence for any difference in learning rate between trichromats and simulated dichromats (see Figure 4). See Table 1 and the Supplementary Statistical Output for all model terms.

**Table 1:** Experiment 1 terms and interactions retained following model simplification of the nightjar data

Model Terms	DF	*F*	*P*
logEdgeDist	1	4399.84	<0.001
firstSlide	1	3203.78	<0.001
playedBefore	1	2629.40	<0.001
slideNumber	1	2192.00	<0.001
viewingCondition	1	91.40	<0.001
logNearPatternDiff	1	0.77	0.381
nearLuminanceDiff	1	0.04	0.845
adultArea	1	31.58	<0.001
nearLuminanceMean	1	2.96	0.085
ageRange	4	270.56	<0.001
viewingCondition:logNearPatternDiff	1	81.99	<0.001
viewingCondition:nearLuminanceDiff	1	111.84	<0.001
viewingCondition:adultArea	1	410.69	<0.001
viewingCondition:nearLuminanceMean	1	130.6758	<0.001

### Experiment 2: plover and courser nests

**Figure 3:**
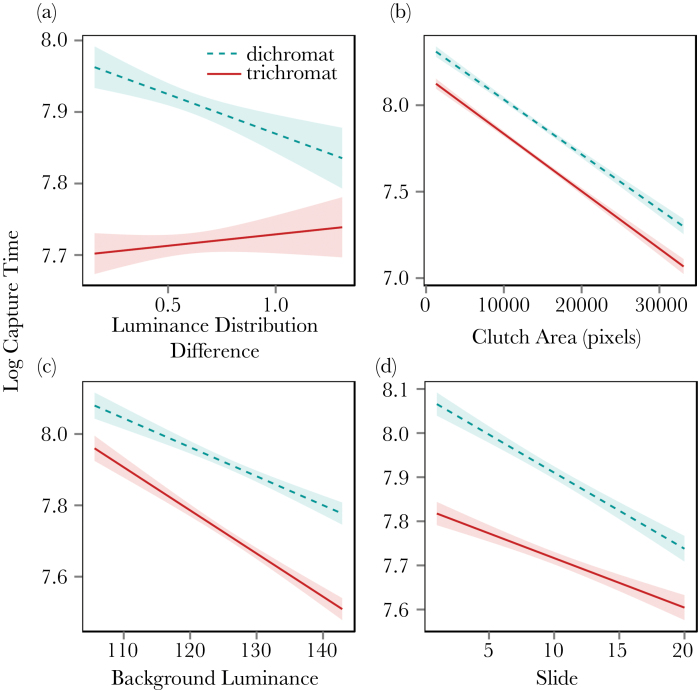
Plots showing the camouflage variables that were found to be affected by trichromatic or simulated dichromatic viewing conditions in the egg searching game (*P*-values for all 4 interactions <0.005). Overall, trichromats had an advantage over simulated dichromats in all circumstances. However, the interactions with slide number (panel d) show dichromats learning to find targets faster than trichromats. Simulated dichromats were more susceptible to changes in luminance difference between the eggs and their backgrounds (panel a), although their success was less dependent on changes in the size of the clutch (paned b), and background luminance (panel c).

**Figure 4:**
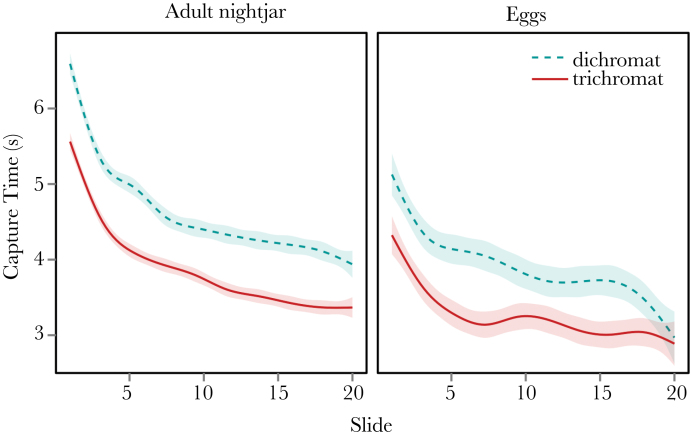
Learning rates in experiment 1 (adult nightjars) and experiment 2 (plover and courser egg clutches). Raw data were plotted with GAM, generalized additive model smoothing, with shaded regions showing standard error. Experiment 1 shows longer initial capture times than experiment 2, but faster learning rates with asymptotes at around slide 15. There were no learning differences detected between trichromats and simulated dichromats in experiment 1; however, simulated dichromats learnt to find targets faster than trichromats in experiment 2 and were still improving at the end of the session.

A total of 22810 successful egg captures were recorded from 1531 game plays (620 naive players). Overall, trichromats found eggs faster than simulated dichromats under all conditions. The simplified model retained 4 interactions, all involving viewing condition as follows: 1) simulated dichromat performance improved with increased luminance distribution difference when compared to trichromats (*t* = 6.66, *F*_1,21174_ = 24.0, *P* < 0.005, i.e. the slope of dichromats in Figure 3a is negative, whereas it is flat for trichromats). As an example of effect size, prey in the lowest 10th quantile of luminance distribution differences, had a predation risk 36% greater when viewed as a trichromat than when viewed as a simulated dichromat based on the raw data (23% based on model predictions). 2) Eggs against darker backgrounds were more difficult to find, and simulated dichromats were significantly less affected by background luminance than were trichromats (*t* = −4.10, *F*_1,21174_ = 16.8, *P* < 0.005). 3) Smaller targets were more difficult to find, and simulated dichromats were more affected by target size than were trichromats (*t* = 3.94, *F*_1,21174_ = 20.5, *P* < 0.005). 4) Simulated dichromats learnt to find the target significantly faster than did trichromats (*t* = 3.97, *F*_1,21174_ = 15.4, *P* < 0.005; see Figures 3 and 4). See Figure 3, Table 2 and the Supplementary Statistical Output for all model terms. When the difficulty level of the egg game (i.e., easy or hard) was included in the full model, it was retained in the simplified model but the camouflage results were unchanged (i.e., the same 4 interactions remained in the model; data not shown).

**Table 2:** Experiment 2 terms and interactions retained following model simplification of the plover and courser nest data

Model Terms	DF	*F*	*P*
logEdgeDistance	1	1333.26	<0.001
firstSlide	1	379.15	<0.001
playedBefore	1	459.09	<0.001
ageRange	4	31.47	<0.001
slideNumber	1	115.62	<0.001
viewingCondition	1	44.84	<0.001
wholeLuminanceDiff	1	0.31	0.5775
clutchArea	1	47.80	<0.001
wholeLuminanceMean	1	11.86	<0.001
slide:viewingCondition	1	15.35	<0.001
viewingCondition:wholeLuminanceDiff	1	24.00	<0.001
viewingCondition:clutchArea	1	20.53	<0.001
viewingCondition:wholeLuminanceMean	1	16.821	<0.001

## DISCUSSION

A restricted range of color vision has long been thought to confer potential advantages in camouflage breaking, although evidence for this remains somewhat equivocal, and recent studies that found support for this theory were unable to elucidate which aspects of camouflage were responsible for the effect (Melin et al. 2007; Smith et al. 2012). Here we tested numerous camouflage metrics in order to determine how color vision affects performance in detecting cryptic prey, using citizen science games that attracted thousands of players. The games highlighted substantial differences between the camouflage breaking abilities of trichromats and simulated dichromats when searching for cryptic prey against natural backgrounds, although overall we found that simulated dichromats performed worse than trichromats in our experiments.

Both games demonstrated that larger targets were found more easily, and that they were easier to find against brighter backgrounds, although there were also differences between the results of the 2 games. The effects of luminance distribution difference, for example, were contradictory between games, suggesting that participants may have been adopting different search strategies in each type of game (i.e., when finding nightjars vs. eggs; see Figures 2 and 3), or alternatively that there were qualitative differences in camouflage strategies used by the eggs and nightjars. Nightjars were always photographed side-on, meaning that their overall outline and shape was a consistent cue between images that could have facilitated the formation/learning of a search image (Bond and Kamil 1999). Furthermore, the patterning of nightjar plumage is quite consistent between individuals, meaning that a search for specific patterns could be an effective strategy. In contrast, clutches of eggs varied considerably in the shape, size, and angle of their outlines, and their patterning was highly variable within and between species, perhaps ruling out search-image formation for specific patterns or characteristic outlines. Comparing the learning rates between the 2 experiments supports the hypothesis that qualitative differences in learning could explain our results; Figure 4 shows that learning rates were considerably higher in the nightjar game than the egg game, particularly in the first 5 slides.

In a companion study that measured actual predation rates on these species in the field in Zambia, pattern difference was found to be a significant predictor of nightjar clutch survival against real predators: adults that matched the pattern of their surrounds were less likely to suffer natural predation (Troscianko et al. 2016b). However, the data presented here suggest the opposite effect, since adult nightjars were easier to find by simulated human predators when their pattern matched their background more closely. This counter-intuitive result may support the search-image theory (above), whereby participants learnt to find the most common nightjar shapes and patterns, and this average nightjar template also matched the average background well. Any nightjars that did not match this average search image would then be at an advantage under search-image theory, and may benefit from other cryptic phenomena that do not result in background pattern matching, such as disruptive camouflage in which high contrast patterns break out the animal’s tell-tale outline (Cuthill et al. 2005; Troscianko et al. 2013), or distractive markings may draw the predator’s attention away from its important outline features (Stevens and Merilaita 2009). It is highly unlikely that natural predators would have the opportunity to form such a specific search image for nightjars, because they would encounter them infrequently (certainly considerably more than a few seconds apart), interspersed with other animals, and at different angles and distances, compared to this experiment where angle and distance were fixed. Nevertheless, in line with expectations, simulated dichromat capture times were less affected by pattern difference than trichromats, but simulated dichromats were also less able to find nightjars that had a larger luminance mismatch with their background than were trichromats. Taken together this suggests that simulated dichromats are either more susceptible to non–background-matching effects than trichromats (such as edge disruption), or are better able to detect subtle pattern cues. Further work should determine whether the evolution of different camouflage strategies (such as disruptive or distractive markings) is affected by the color vision properties of predator types exerting the strongest selection.

The egg searching game supported our prediction that simulated dichromats should be less affected than trichromats by background luminance conditions, given dichromats prefer to forage in lower light conditions than their trichromat conspecifics (Yamashita et al. 2005; Melin et al. 2007; Caine et al. 2010, see Figure 3c). However, their overall performance was still worse than that of trichromats across all luminance levels, and this effect was not observed in the adult nightjar game. Simulated dichromats in the egg game were also less able to detect eggs with more subtle luminance differences with their background (Figure 3a). The lighting conditions in studies that found dichromats forage more in lower light than their trichromat conspecifics (Yamashita et al. 2005; Melin et al. 2007; Caine et al. 2010) might also correlate with a number of other variables, from physical habitat properties to social or behavioral differences. For example, understorey habitats will have proportionately more green wavelengths than the light above the canopy, possibly making the red–green opponent channel less sensitive under the canopy than above. The lighting intensity differences under sunlight in the wild will also be higher than those recreated in our experiment by computer screens. The limitations of our computer game experiment (using uncalibrated displays) prevented us from investigating these subtle color differences and extreme lighting intensity differences, so further work would be required to ascertain whether the lighting differences in di/trichromat performance are caused by overall luminous intensity differences, shifts in spectral radiance, or other habitat-related variables such as the dynamic range of a scene or color of shadows.

Our egg game highlighted a difference in learning rates between viewing conditions, with simulated dichromats learning to find the eggs faster than trichromats. To our knowledge these are the first data to demonstrate a difference in camouflage-breaking learning rates dependent on color vision. Nevertheless, it remains to be seen whether the effects we have found using simulated dichromats reflect those of natural dichromats that have had their visual systems adapted for 2 color channels all their lives (e.g., see Dain and King-Smith 1981, Sharpe et al. 2006). It is possible that simulating reduced color vision for a short period in trichromats results in qualitative differences in visual processing compared to that of natural dichromats, although simulated viewing conditions have proved valuable in the past (Melin et al. 2013). Furthermore, even if observers can to an extent compensate for simulated differences in visual dimensions, our treatments essentially change the amount of visual information available in the visual scene regardless of neural processing differences. Camouflage strategies and contrast levels are known to affect learning rates in touch-screen experiments with humans, who learnt to find distractive markings faster over successive slides than they learnt to find background-matching prey (Stevens et al. 2013). Humans also learnt to find prey with high-contrast markings associated with disruptive patterns more quickly over time than they learnt to find background-matching prey (Troscianko et al. 2013). However, those experiments did not investigate any colorimetric variables that are likely to be important when investigating the differences between color vision systems. Learning to find cryptic targets faster with restricted color vision could arise if participants learnt to adapt to the loss of some color information, in which case we would predict that simulated dichromats could at best converge with trichromat performance, but not exceed it. Alternatively, the loss of potentially distracting color information (previously shown to affect overall performance in some search tasks: Morgan et al. 1992; Saito et al. 2005; Saito et al. 2006) could have allowed subjects to adopt a search strategy that enables faster learning, in which case simulated dichromats could out-perform trichromats over time at certain tasks. Figure 4 shows that after the 20 slides of this experiment, simulated dichromats were performing on a par with trichromats searching for eggs. Experiments with a larger number of slides would be required to determine whether simulated dichromats would continue to improve further and eventually out-perform trichromats. Nevertheless, these data do demonstrate that humans can rapidly learn to perform well in camouflage breaking tasks that have color information removed. These hypotheses should be tested in further experiments to investigate learning rates in a wide range of cryptic, ecologically relevant stimuli that demonstrate large natural variation in background types and camouflage strategies (for example, disruptive, distractive, and background matching prey types). It would also be interesting to investigate whether there is a difference in specialist and generalist search strategies between viewing conditions. For example, can dichromats attend to more general cues to break camouflage, rather than relying on specific search images to find prey? Taken together, this study suggests that color perception interacts with camouflage breaking in complex ways, which could help explain why color vision with just 2 receptor types is so widespread in nature (Cronin et al. 2014).

## SUPPLEMENTARY MATERIAL

Supplementary data are available at *Behavioral Ecology* online.

## FUNDING

This work was supported by Biotechnology and Biological Sciences Research Council (BBSRC) grants BB/L017709/1 and BB/J018309/1 to M.S., and C.N.S. was funded by a Royal Society Dorothy Hodgkin Fellowship, a BBSRC David Phillips Fellowship (BB/J014109/1) and the DST-NRF Centre of Excellence at the FitzPatrick Institute.

## Supplementary Material

Fiery_neck_di_tri

Statistical_Output
