# Supplementary figures and images for: Relative advantages of dichromatic and trichromatic color vision in camouflage breaking
Source: Behav Ecol. 2017 Feb 4;28(2):556–64. doi: 10.1093/beheco/arw185 (PMC5873837; doi:10.1093/beheco/arw185)

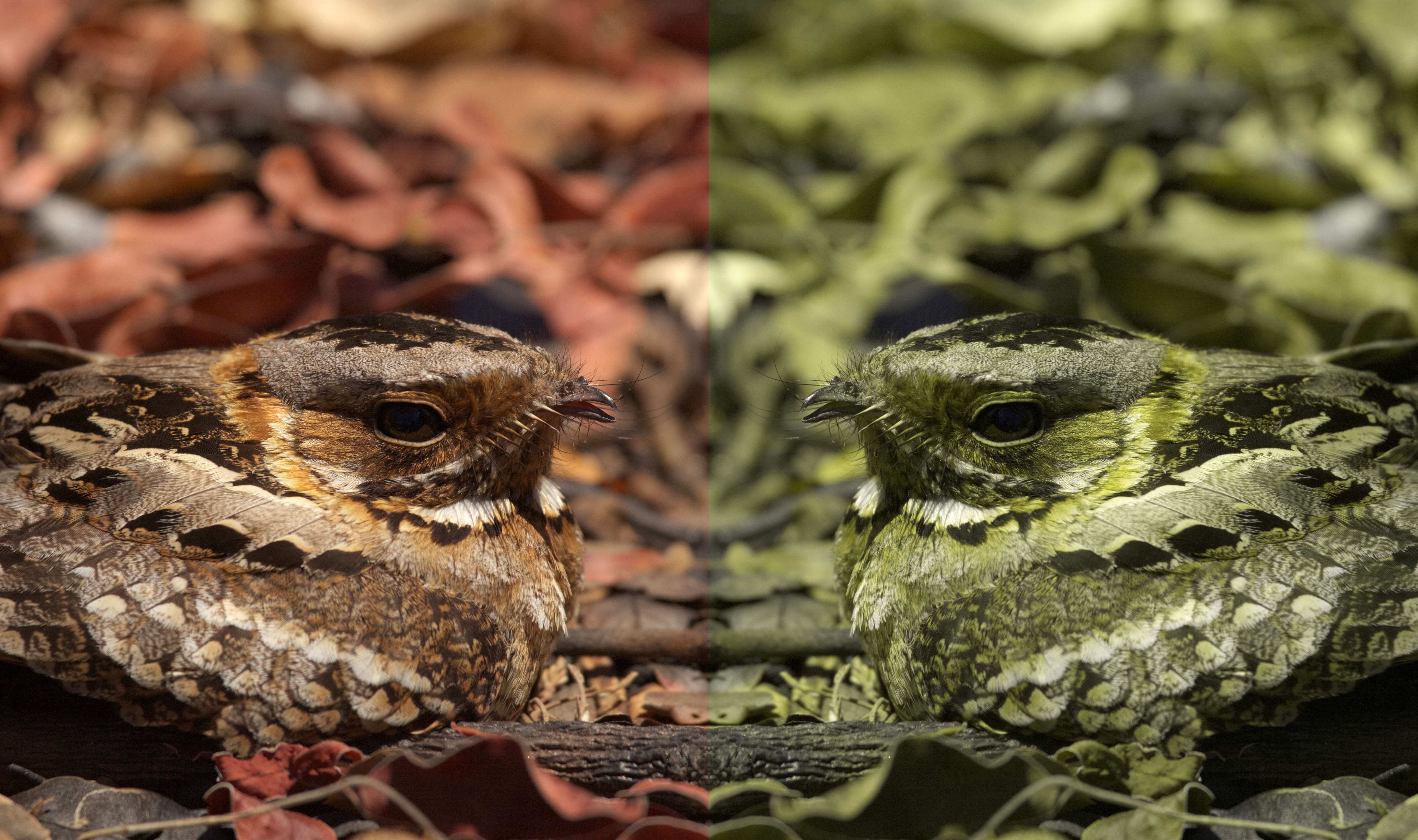

Supplement: Fiery_neck_di_tri [file arw185_suppl_fiery_neck_di_tri.jpeg]
