# Supplementary material for: Relative advantages of dichromatic and trichromatic color vision in camouflage breaking
Source: Behav Ecol. 2017 Feb 4;28(2):556–64. doi: 10.1093/beheco/arw185 (PMC5873837; doi:10.1093/beheco/arw185)
Supplement: Statistical_Output [file arw185_suppl_statistical_output.doc]

-----------------------------FULL MODEL FOR FINDING ADULT NIGHTJARS-----------------------

Linear mixed model fit by REML

t-tests use Satterthwaite approximations to degrees of freedom ['lmerMod']

Formula: log(time) ~ logEdgeDist + firstSlide + played_before + njspecies + (slide + species + logNearPatternDiff + nearLumDiff + adultarea +

nightjarContrast + nearlumMean + nearContrast + age_range)^2 + (1 | individual) + (1 | nest)

Data: adultdata

REML criterion at convergence: 250394.5

Scaled residuals:

Min 1Q Median 3Q Max

-6.1936 -0.6550 -0.1182 0.5410 4.4576

Random effects:

Groups Name Variance Std.Dev.

individual (Intercept) 0.13836 0.3720

nest (Intercept) 0.07934 0.2817

Residual 0.32220 0.5676

Number of obs: 135968, groups: individual, 9926; nest, 39

Fixed effects:

Estimate Std. Error df t value Pr(>|t|)

(Intercept) 7.148e+00 1.259e+01 1.500e+01 0.568 0.578560

logEdgeDist -4.049e-01 6.113e-03 1.290e+05 -66.246 < 2e-16 ***

firstSlide1 2.536e-01 7.123e-03 1.310e+05 35.606 < 2e-16 ***

played_before1 -4.112e-01 8.870e-03 8.644e+03 -46.360 < 2e-16 ***

njspeciesCP 9.121e-02 2.921e-01 1.500e+01 0.312 0.759128

njspeciesMV 8.548e-02 2.103e-01 1.500e+01 0.406 0.690153

slide -3.675e-02 4.883e-03 1.313e+05 -7.526 5.26e-14 ***

speciesmonkey 1.842e-01 7.875e-02 2.776e+04 2.339 0.019322 *

logNearPatternDiff 3.307e+00 2.694e+00 1.500e+01 1.227 0.238431

nearLumDiff 2.025e+00 5.706e+00 1.500e+01 0.355 0.727503

adultarea -2.920e-05 1.268e-04 1.500e+01 -0.230 0.820981

nightjarContrast -2.356e+01 1.687e+01 1.500e+01 -1.396 0.182740

nearlumMean -2.733e-02 4.460e-02 1.500e+01 -0.613 0.549110

nearContrast 2.584e+01 1.683e+01 1.500e+01 1.535 0.145351

age_range2 -4.888e-01 2.611e-01 1.358e+05 -1.872 0.061155 .

age_range3 -5.395e-01 2.016e-01 1.358e+05 -2.677 0.007440 **

age_range4 -4.956e-01 2.064e-01 1.358e+05 -2.401 0.016364 *

age_range5 -4.158e-01 2.100e-01 1.358e+05 -1.980 0.047659 *

slide:speciesmonkey 2.036e-03 5.582e-04 1.324e+05 3.647 0.000265 ***

slide:logNearPatternDiff -1.670e-03 8.216e-04 1.297e+05 -2.033 0.042065 *

slide:nearLumDiff 6.788e-04 1.765e-03 1.296e+05 0.384 0.700612

slide:adultarea 7.959e-08 3.234e-08 1.297e+05 2.461 0.013857 *

slide:nightjarContrast 3.185e-03 4.120e-03 1.296e+05 0.773 0.439486

slide:nearlumMean 8.808e-05 1.751e-05 1.296e+05 5.030 4.92e-07 ***

slide:nearContrast 1.776e-02 5.850e-03 1.296e+05 3.037 0.002392 **

slide:age_range2 4.978e-03 2.854e-03 1.348e+05 1.744 0.081175 .

slide:age_range3 4.255e-03 2.207e-03 1.354e+05 1.928 0.053879 .

slide:age_range4 4.099e-03 2.259e-03 1.353e+05 1.814 0.069624 .

slide:age_range5 3.199e-03 2.294e-03 1.353e+05 1.394 0.163309

speciesmonkey:logNearPatternDiff -8.839e-02 9.538e-03 1.280e+05 -9.266 < 2e-16 ***

speciesmonkey:nearLumDiff -1.757e-01 2.039e-02 1.280e+05 -8.617 < 2e-16 ***

speciesmonkey:adultarea 7.407e-06 3.701e-07 1.281e+05 20.014 < 2e-16 ***

speciesmonkey:nightjarContrast -6.536e-02 4.739e-02 1.280e+05 -1.379 0.167818

speciesmonkey:nearlumMean 1.122e-03 2.024e-04 1.280e+05 5.542 3.00e-08 ***

speciesmonkey:nearContrast -1.920e-01 6.688e-02 1.280e+05 -2.871 0.004086 **

speciesmonkey:age_range2 2.531e-02 8.148e-02 9.586e+03 0.311 0.756076

speciesmonkey:age_range3 -4.693e-02 6.141e-02 1.005e+04 -0.764 0.444795

speciesmonkey:age_range4 -1.966e-02 6.315e-02 9.967e+03 -0.311 0.755552

speciesmonkey:age_range5 -5.728e-02 6.404e-02 9.957e+03 -0.894 0.371137

logNearPatternDiff:nearLumDiff -1.520e+00 1.283e+00 1.500e+01 -1.185 0.254376

logNearPatternDiff:adultarea 4.562e-06 2.387e-05 1.500e+01 0.191 0.850945

logNearPatternDiff:nightjarContrast 5.102e+00 3.363e+00 1.500e+01 1.517 0.149897

logNearPatternDiff:nearlumMean -7.964e-04 8.730e-03 1.500e+01 -0.091 0.928510

logNearPatternDiff:nearContrast -1.050e+01 4.787e+00 1.500e+01 -2.193 0.044321 *

logNearPatternDiff:age_range2 1.401e-02 4.666e-02 1.290e+05 0.300 0.763880

logNearPatternDiff:age_range3 7.681e-03 3.551e-02 1.296e+05 0.216 0.828764

logNearPatternDiff:age_range4 -3.776e-02 3.644e-02 1.295e+05 -1.036 0.300042

logNearPatternDiff:age_range5 -1.754e-02 3.701e-02 1.295e+05 -0.474 0.635548

nearLumDiff:adultarea 2.418e-05 6.865e-05 1.500e+01 0.352 0.729511

nearLumDiff:nightjarContrast 1.123e+00 5.339e+00 1.500e+01 0.210 0.836144

nearLumDiff:nearlumMean 7.491e-03 2.301e-02 1.500e+01 0.325 0.749276

nearLumDiff:nearContrast 2.818e+00 5.381e+00 1.500e+01 0.524 0.608070

nearLumDiff:age_range2 1.940e-01 9.982e-02 1.290e+05 1.944 0.051932 .

nearLumDiff:age_range3 2.077e-01 7.655e-02 1.294e+05 2.714 0.006654 **

nearLumDiff:age_range4 2.341e-01 7.855e-02 1.293e+05 2.981 0.002877 **

nearLumDiff:age_range5 2.637e-01 7.966e-02 1.293e+05 3.311 0.000931 ***

adultarea:nightjarContrast -1.153e-04 1.632e-04 1.500e+01 -0.706 0.490732

adultarea:nearlumMean -9.247e-08 5.345e-07 1.500e+01 -0.173 0.864938

adultarea:nearContrast 1.129e-04 1.869e-04 1.500e+01 0.604 0.554711

adultarea:age_range2 -1.684e-06 1.821e-06 1.291e+05 -0.925 0.355179

adultarea:age_range3 -2.330e-06 1.383e-06 1.295e+05 -1.685 0.092007 .

adultarea:age_range4 -2.133e-06 1.421e-06 1.295e+05 -1.501 0.133313

adultarea:age_range5 -2.277e-06 1.441e-06 1.295e+05 -1.580 0.114126

nightjarContrast:nearlumMean 3.190e-02 8.140e-02 1.500e+01 0.392 0.700651

nightjarContrast:nearContrast 8.177e+00 1.283e+01 1.500e+01 0.638 0.533298

nightjarContrast:age_range2 -1.834e-01 2.370e-01 1.291e+05 -0.774 0.439142

nightjarContrast:age_range3 -1.064e-01 1.827e-01 1.294e+05 -0.582 0.560388

nightjarContrast:age_range4 -2.217e-01 1.872e-01 1.293e+05 -1.184 0.236460

nightjarContrast:age_range5 -2.236e-01 1.902e-01 1.293e+05 -1.175 0.239807

nearlumMean:nearContrast 1.116e-02 7.378e-02 1.500e+01 0.151 0.881809

nearlumMean:age_range2 -2.587e-04 1.009e-03 1.288e+05 -0.256 0.797640

nearlumMean:age_range3 -2.337e-04 7.753e-04 1.289e+05 -0.301 0.763092

nearlumMean:age_range4 7.069e-04 7.944e-04 1.288e+05 0.890 0.373522

nearlumMean:age_range5 1.186e-03 8.068e-04 1.288e+05 1.470 0.141577

nearContrast:age_range2 4.467e-01 3.329e-01 1.291e+05 1.342 0.179702

nearContrast:age_range3 3.983e-01 2.577e-01 1.293e+05 1.545 0.122229

nearContrast:age_range4 7.198e-01 2.643e-01 1.293e+05 2.723 0.006469 **

nearContrast:age_range5 8.511e-01 2.684e-01 1.293e+05 3.171 0.001519 **

---

Signif. codes: 0 ‘***’ 0.001 ‘**’ 0.01 ‘*’ 0.05 ‘.’ 0.1 ‘ ’ 1

------------------------SIMPLIFIED MODEL FOR FINDING ADULT NIGHTJARS---------------------

Linear mixed model fit by REML ['lmerMod']

Formula: log(time) ~ logEdgeDist + firstSlide + played_before + slide + species + logNearPatternDiff + nearLumDiff + adultarea +

nearlumMean + age_range + (1 | individual) + (1 | nest) + species:logNearPatternDiff + species:nearLumDiff + species:adultarea +

species:nearlumMean

Data: adultdata

REML criterion at convergence: 250049.4

Scaled residuals:

Min 1Q Median 3Q Max

-6.1523 -0.6557 -0.1188 0.5424 4.4697

Random effects:

Groups Name Variance Std.Dev.

individual (Intercept) 0.13813 0.3717

nest (Intercept) 0.09272 0.3045

Residual 0.32250 0.5679

Number of obs: 135968, groups: individual, 9926; nest, 39

Fixed effects:

Estimate Std. Error t value

(Intercept) 1.107e+01 3.479e-01 31.82

logEdgeDist -4.049e-01 6.114e-03 -66.22

firstSlide1 2.533e-01 7.121e-03 35.57

played_before1 -4.111e-01 8.855e-03 -46.43

slide -1.403e-02 2.996e-04 -46.84

speciesmonkey -3.388e-02 2.513e-02 -1.35

logNearPatternDiff 3.200e-01 1.207e-01 2.65

nearLumDiff 1.063e-01 2.187e-01 0.49

adultarea -3.625e-05 5.593e-06 -6.48

nearlumMean -4.819e-03 2.262e-03 -2.13

age_range2 -2.309e-01 3.978e-02 -5.80

age_range3 -3.261e-01 2.970e-02 -10.98

age_range4 -1.938e-01 3.061e-02 -6.33

age_range5 5.058e-02 3.108e-02 1.63

speciesmonkey:logNearPatternDiff -9.645e-02 7.899e-03 -12.21

speciesmonkey:nearLumDiff -1.671e-01 1.402e-02 -11.92

speciesmonkey:adultarea 7.565e-06 3.552e-07 21.30

speciesmonkey:nearlumMean 1.716e-03 1.501e-04 11.43

Correlation of Fixed Effects:

(Intr) lgEdgD frstS1 plyd_1 slide spcsmn lgNrPD nrLmDf adultr nrlmMn ag_rn2 ag_rn3 ag_rn4 ag_rn5 sp:NPD spc:LD spcsm:

logEdgeDist -0.101

firstSlide1 -0.005 -0.002

played_bfr1 -0.011 -0.002 -0.011

slide -0.008 -0.002 0.372 -0.006

speciesmnky -0.035 0.006 -0.002 -0.066 -0.005

lgNrPttrnDf -0.769 0.000 0.000 0.000 0.000 0.023

nearLumDiff 0.118 0.000 0.000 0.000 0.000 -0.003 -0.186

adultarea -0.156 0.001 -0.001 0.000 0.001 0.004 -0.208 0.100

nearlumMean -0.248 0.000 0.000 0.000 0.000 0.009 -0.262 -0.393 0.096

age_range2 -0.062 0.000 0.000 0.028 -0.004 0.001 0.000 0.000 0.000 0.000

age_range3 -0.083 0.001 0.003 0.044 -0.008 0.009 0.000 0.000 0.000 0.000 0.720

age_range4 -0.081 0.000 0.003 0.050 -0.009 0.009 0.000 0.000 0.000 0.000 0.699 0.938

age_range5 -0.080 0.001 0.003 0.067 -0.008 0.002 0.000 0.000 0.000 0.000 0.689 0.924 0.897

spcsmnk:NPD 0.026 -0.004 -0.001 0.002 0.002 -0.742 -0.033 0.005 0.008 0.007 0.000 0.001 0.001 0.001

spcsmnky:LD -0.004 0.001 0.002 -0.002 0.001 0.148 0.005 -0.033 -0.002 0.014 -0.001 0.000 0.000 0.000 -0.190

spcsmnky:dl 0.004 0.002 0.003 -0.002 -0.005 -0.104 0.008 -0.002 -0.033 -0.004 0.002 0.001 0.001 0.001 -0.248 0.076

spcsmnky:nM 0.011 -0.004 0.005 0.000 0.002 -0.336 0.007 0.013 -0.004 -0.033 0.002 0.001 0.000 0.001 -0.160 -0.422 0.099

--------------------------------FULL MODEL FOR FINDING NESTS------------------------------

Linear mixed model fit by REML

t-tests use Satterthwaite approximations to degrees of freedom ['lmerMod']

Formula: log(time) ~ logEdgeDist + firstSlide + played_before + age_range + logWholePatternDiff + (slide + visualSystem + wholeLumDiff +

clutcharea + clutchContrast + wholelumMean + wholeContrast)^2 + (1 | individual) + (1 | nest)

Data: eggdata

REML criterion at convergence: 38354.2

Scaled residuals:

Min 1Q Median 3Q Max

-3.6437 -0.6264 -0.1292 0.4808 4.8482

Random effects:

Groups Name Variance Std.Dev.

individual (Intercept) 0.1089 0.3299

nest (Intercept) 0.1027 0.3204

Residual 0.2704 0.5200

Number of obs: 22810, groups: individual, 1531; nest, 88

Fixed effects:

Estimate Std. Error df t value Pr(>|t|)

(Intercept) 6.779e+00 6.639e+00 7.000e+01 1.021 0.310710

logEdgeDist -5.023e-01 1.378e-02 2.161e+04 -36.454 < 2e-16 ***

firstSlide1 2.243e-01 1.605e-02 2.178e+04 13.981 < 2e-16 ***

played_before1 -3.645e-01 2.076e-02 1.340e+03 -17.554 < 2e-16 ***

age_range2 -7.193e-01 9.269e-02 1.332e+03 -7.760 1.69e-14 ***

age_range3 -5.323e-01 8.569e-02 1.357e+03 -6.212 6.96e-10 ***

age_range4 -5.177e-01 8.761e-02 1.356e+03 -5.909 4.36e-09 ***

age_range5 -3.493e-01 8.950e-02 1.353e+03 -3.903 9.95e-05 ***

logWholePatternDiff 3.693e-01 1.013e-01 7.000e+01 3.646 0.000506 ***

slide 1.907e-03 1.871e-02 2.173e+04 0.102 0.918805

visualSystemmonkey -5.791e-01 2.130e-01 2.187e+04 -2.719 0.006553 **

wholeLumDiff 1.558e+01 5.137e+00 7.000e+01 3.032 0.003406 **

clutcharea 2.869e-04 2.137e-04 6.900e+01 1.342 0.183889

clutchContrast -3.804e+01 1.337e+01 7.000e+01 -2.845 0.005814 **

wholelumMean 2.578e-02 4.129e-02 7.000e+01 0.624 0.534420

wholeContrast 2.576e+01 9.747e+00 7.000e+01 2.643 0.010144 *

slide:visualSystemmonkey 4.762e-03 1.242e-03 2.202e+04 3.833 0.000127 ***

slide:wholeLumDiff 4.534e-03 2.764e-03 2.172e+04 1.640 0.100987

slide:clutcharea 1.980e-08 1.095e-07 2.170e+04 0.181 0.856428

slide:clutchContrast -3.368e-02 8.768e-03 2.172e+04 -3.842 0.000123 ***

slide:wholelumMean -9.121e-05 1.134e-04 2.171e+04 -0.804 0.421211

slide:wholeContrast 3.110e-02 1.744e-02 2.179e+04 1.783 0.074537 .

visualSystemmonkey:wholeLumDiff 1.910e-01 3.152e-02 2.153e+04 6.059 1.40e-09 ***

visualSystemmonkey:clutcharea 4.393e-06 1.247e-06 2.153e+04 3.523 0.000427 ***

visualSystemmonkey:clutchContrast 3.915e-02 1.024e-01 2.188e+04 0.382 0.702198

visualSystemmonkey:wholelumMean -5.018e-05 1.290e-03 2.158e+04 -0.039 0.968971

visualSystemmonkey:wholeContrast 6.815e-01 1.996e-01 2.165e+04 3.414 0.000640 ***

wholeLumDiff:clutcharea -2.944e-05 3.050e-05 7.000e+01 -0.965 0.337827

wholeLumDiff:clutchContrast 2.984e+00 2.118e+00 7.000e+01 1.409 0.163316

wholeLumDiff:wholelumMean -8.585e-02 3.104e-02 7.000e+01 -2.766 0.007271 **

wholeLumDiff:wholeContrast -1.898e+01 4.970e+00 7.000e+01 -3.819 0.000287 ***

clutcharea:clutchContrast 2.812e-04 7.975e-05 7.000e+01 3.526 0.000747 ***

clutcharea:wholelumMean -2.104e-06 1.336e-06 6.900e+01 -1.575 0.119839

clutcharea:wholeContrast -4.815e-04 1.834e-04 7.000e+01 -2.626 0.010610 *

clutchContrast:wholelumMean 2.168e-01 8.334e-02 7.000e+01 2.601 0.011324 *

clutchContrast:wholeContrast 1.402e+01 1.152e+01 7.000e+01 1.217 0.227788

wholelumMean:wholeContrast -1.154e-01 6.270e-02 7.000e+01 -1.840 0.070002 .

---

Signif. codes: 0 ‘***’ 0.001 ‘**’ 0.01 ‘*’ 0.05 ‘.’ 0.1 ‘ ’ 1

-----------------------------SIMPLIFIED MODEL FOR FINDING NESTS---------------------------

Linear mixed model fit by REML ['lmerMod']

Formula: log(time) ~ logEdgeDist + firstSlide + played_before + age_range + slide + visualSystem + wholeLumDiff + clutcharea + wholelumMean +

(1 | individual) + (1 | nest) + slide:visualSystem + visualSystem:wholeLumDiff + visualSystem:clutcharea + visualSystem:wholelumMean

Data: eggdata

REML criterion at convergence: 38293.6

Scaled residuals:

Min 1Q Median 3Q Max

-3.6237 -0.6273 -0.1292 0.4820 4.8029

Random effects:

Groups Name Variance Std.Dev.

individual (Intercept) 0.1091 0.3303

nest (Intercept) 0.1787 0.4227

Residual 0.2708 0.5204

Number of obs: 22810, groups: individual, 1531; nest, 88

Fixed effects:

Estimate Std. Error t value

(Intercept) 1.451e+01 6.644e-01 21.84

logEdgeDist -5.025e-01 1.378e-02 -36.45

firstSlide1 2.232e-01 1.605e-02 13.91

played_before1 -3.619e-01 2.078e-02 -17.41

age_range2 -7.211e-01 9.277e-02 -7.77

age_range3 -5.298e-01 8.577e-02 -6.18

age_range4 -5.139e-01 8.769e-02 -5.86

age_range5 -3.479e-01 8.958e-02 -3.88

slide -9.651e-03 9.200e-04 -10.49

visualSystemmonkey 9.081e-02 1.126e-01 0.81

wholeLumDiff -1.814e-01 1.868e-01 -0.97

clutcharea -5.190e-05 7.151e-06 -7.26

wholelumMean -1.608e-02 5.225e-03 -3.08

slide:visualSystemmonkey 4.928e-03 1.242e-03 3.97

visualSystemmonkey:wholeLumDiff 1.967e-01 2.953e-02 6.66

visualSystemmonkey:clutcharea 4.539e-06 1.152e-06 3.94

visualSystemmonkey:wholelumMean -3.583e-03 8.735e-04 -4.10

Correlation of Fixed Effects:

(Intr) lgEdgD frstS1 plyd_1 ag_rn2 ag_rn3 ag_rn4 ag_rn5 slide vslSys whlLmD cltchr whllmM sld:vS vsS:LD vslSy:

logEdgeDist -0.119

firstSlide1 -0.006 -0.005

played_bfr1 -0.040 -0.004 -0.001

age_range2 -0.111 0.002 0.001 -0.055

age_range3 -0.130 0.000 0.000 0.040 0.905

age_range4 -0.128 0.001 0.000 0.040 0.888 0.960

age_range5 -0.122 -0.002 -0.001 0.034 0.871 0.938 0.920

slide -0.010 -0.008 0.273 -0.006 -0.001 -0.003 -0.004 -0.003

vslSystmmnk -0.089 0.015 0.005 -0.030 -0.020 -0.013 -0.018 -0.016 0.067

wholeLumDff 0.020 0.000 0.000 0.000 0.000 0.000 0.000 0.000 0.000 -0.006

clutcharea -0.199 0.000 -0.001 0.020 -0.003 0.003 0.004 0.001 0.000 0.027 0.249

wholelumMen -0.951 0.000 0.001 0.020 -0.003 0.003 0.004 0.002 -0.002 0.084 -0.229 0.017

sld:vslSyst 0.006 0.010 -0.001 0.002 -0.002 0.005 0.003 0.002 -0.686 -0.095 -0.001 -0.001 0.001

vslSystm:LD -0.007 0.008 0.003 -0.003 0.001 -0.001 -0.001 -0.001 0.000 0.067 -0.081 -0.013 0.022 0.001

vslSystmmn: 0.028 0.000 0.009 -0.003 -0.002 0.002 0.003 0.002 -0.007 -0.299 -0.013 -0.084 -0.014 0.006 0.183

vslSystmm:M 0.087 -0.018 -0.007 -0.005 -0.005 0.002 0.004 0.001 0.002 -0.952 0.021 -0.013 -0.088 -0.007 -0.257 0.134
